# Manipulating Living Cells to Construct a 3D Single-Cell Assembly without an Artificial Scaffold

**DOI:** 10.3390/polym9080319

**Published:** 2017-07-30

**Authors:** Aoi Yoshida, Shoto Tsuji, Hiroaki Taniguchi, Takahiro Kenmotsu, Koichiro Sadakane, Kenichi Yoshikawa

**Affiliations:** 1 Faculty of Life and Medical Sciences, Doshisha University, Kyoto 610-0394, Japan; aoi.yoshida342@gmail.com (A.Y.); t.shoto.bmm1109@gmail.com (S.T.); tkenmots@mail.doshisha.ac.jp (T.K.); 2 The Institute of Genetics and Animal Breeding, Polish Academy of Sciences, Postepu 36A, Jastrzebiec, 05-552 Magdalenka, Poland; h.taniguchi@ighz.pl

**Keywords:** 3D cellular assembly, optical tweezers, crowding effect, depletion effect, remote control

## Abstract

Artificial scaffolds such as synthetic gels or chemically-modified glass surfaces that have often been used to achieve cell adhesion are xenobiotic and may harm cells. To enhance the value of cell studies in the fields of regenerative medicine and tissue engineering, it is becoming increasingly important to create a cell-friendly technique to promote cell–cell contact. In the present study, we developed a novel method for constructing stable cellular assemblies by using optical tweezers in a solution of a natural hydrophilic polymer, dextran. In this method, a target cell is transferred to another target cell to make cell–cell contact by optical tweezers in a culture medium containing dextran. When originally non-cohesive cells are held in contact with each other for a few minutes under laser trapping, stable cell–cell adhesion is accomplished. This method for creating cellular assemblies in the presence of a natural hydrophilic polymer may serve as a novel next-generation 3D single-cell assembly system with future applications in the growing field of regenerative medicine.

## 1. Introduction

Stem-cell-based tissue engineering has emerged as a promising approach for the treatment of intractable diseases [1,2]. To enhance the value of cell studies in the fields of regenerative medicine and tissue engineering, it is becoming increasingly important to create a cell-friendly technique for three-dimensional (3D) cellular assembly [3]. The development of an efficient, minimally-invasive cellular system capable of 3D assembly within an in vitro or in vivo micro environment has been challenging. We recently reported a novel system that could generate a single-cell-based 3D assembly of cells using polyethylene glycol (PEG) by establishing stable cell–cell contact even after the cell assembly was transferred to a PEG-free solution [4]. While synthetic polymers and hydrogels, including PEG, have defined chemical structures and stable physicochemical properties [5,6,7], they are foreign material to cells [8]. It has been reported that PEG promotes cell–cell fusion, and that it has non-negligible effects on the structure and function of living cells in a culture medium [9,10]. Recent trials aimed at producing cellular scaffolds using natural polymers including gelatin, chitosan, dextran, alginate and collagen have been gaining significant momentum [11,12,13,14,15,16]. However, while these natural polymers have also been used to promote cellular adhesion within these cell structures in a non-selective manner, further improvements in hydrogel biocompatibility and adaptability for handling 3D cellular assembly systems are needed.

Dextrans are polysaccharides that contain a linear backbone of α-linked d-glucopyranosyl repeating units [17]. Several studies have implicated dextrans in 3D cellular assembly [18,19]. In this regard, cross-linking glycidyl methacrylate derivatized dextran and dithiothreitol under physiological conditions allows 3D encapsulation of rat bone marrow mesenchymal stem cells (BM-MSCs) and NIH/3T3 fibroblasts; while maintaining high viability [20]. Although it has been proposed that dextran can be used for 3D cellular assembly in a less toxic environment, nearly all of the studies of dextran-induced 3D cellular assembly reported thus far have used a 3D mass cell culture system [21,22]. We and others have recently developed an efficient procedure for constructing cellular assemblies by arranging desired cells at desired positions using optical tweezers in a PEG solution [4,23]. The aim of the present study was to modify the aforementioned PEG protocol to establish a 3D single-cell-based manipulation system that uses a less invasive method of cellular assembly involving optical tweezers and dextran as a natural polymer. This dextran-based method may serve as a novel next-generation 3D single-cell assembling system with future applications in the growing field of regenerative medicine. 

## 2. Materials and Methods

### 2.1. Cell Culture

NAMRU mouse mammary gland epithelial cells (NMuMG cells) were cultured in Dulbecco’s modified Eagle’s medium (DMEM) (Wako Pure Chem. Inc., Osaka, Japan) supplemented with 10% fetal bovine serum (FBS) (Cell Culture Biosci., Nichirei Biosci. Inc., Tokyo, Japan), 40 μg/mL streptomycin, and 40 units/mL penicillin (Life Tech. Corp., Carlsbad, CA, USA). Neuro2A cells were cultured in Dulbecco’s modified Eagle’s medium supplemented with 10% FBS (Atlas Biological., Fort Collins, CO, USA), 1% non-essential amino acids, 100 U/mL penicillin and 10 μg/mL streptomycin (Wako Pure Chem. Inc., Osaka, Japan). The cells were incubated at 37 °C in a humidified atmosphere of 5% CO_2_. Sub-confluent cells were harvested with trypsin (0.25% Trypsin– EDTA (1X)) (Life Tech. Corp., Carlsbad, CA, USA) and cryopreserved with CELLBANKER1 (Nippon Zenyaku Kogyo, Koriyama, Japan). For preparation of the polymer solution, we used dextran (DEX) (200,000; molecular biology-grade, Wako Pure Chem. Inc., Osaka, Japan). We prepared DMEM solution containing 10–50 mg/mL of DEX. 

### 2.2. Single-Beam Optical Tweezers

Optical trapping with single laser beam was carried out using an inverted microscope (TE-300, Nikon) equipped with a Charge Coupled Device (CCD) camera (WAT-120N, Watec Co. Ltd., Tsuruoka, Japan). A 1064 nm Continuous Wave (CW) laser beam (Spectra Physics, Santa Clara, CA, USA) was introduced into the microscope, and focused into the sample through an oil-immersed objective lens (100×, N.A. = 1.3). The laser power at the focal point was set between 42 and 84 mW. All experiments were carried out at room temperature, i.e., 25 °C.

### 2.3. Double-Beam Optical Tweezers

Optical trapping with a double laser beam was carried out using a commercial optical tweezer instrument (NanoTracker 2, JPK Instruments, Berlin, Germany), which is constructed on an inverted microscope (IX71, Olympus, Tokyo, Japan) equipped with a CCD camera (DFK 31AF03, The Imaging Source, Taipei, Taiwan). A 1064 nm CW laser beam was split into two beams by a polarization beam splitter, and both beams were focused into the sample through a water-immersed objective lens (60×, N.A. = 1.2) for independent trapping. One of these focal points can be moved by using a piezo-mirror. The laser power at each focal point was set between 40 and 130 mW. All experiments were carried out at room temperature, i.e., 25 °C.

### 2.4. Viscosity Measurement

The kinetic viscosities of the dextran and polyethylene glycol were measured using a vibrational viscometer (SV-10, A&D Company, Tokyo, Japan) at room temperature (between 19 and 23 °C). The measurement time for each mixture was 60 s.

### 2.5. Cell Viability Assay

The NMuMG cells were cultured in a 6-well dish (Thermo Fisher Scientific, Waltham, MA, USA) and treated with 10–40 mg/mL of DEX. After 24 h of culture, cell viability was verified by trypan blue staining.

## 3. Results

**Figure 1:**
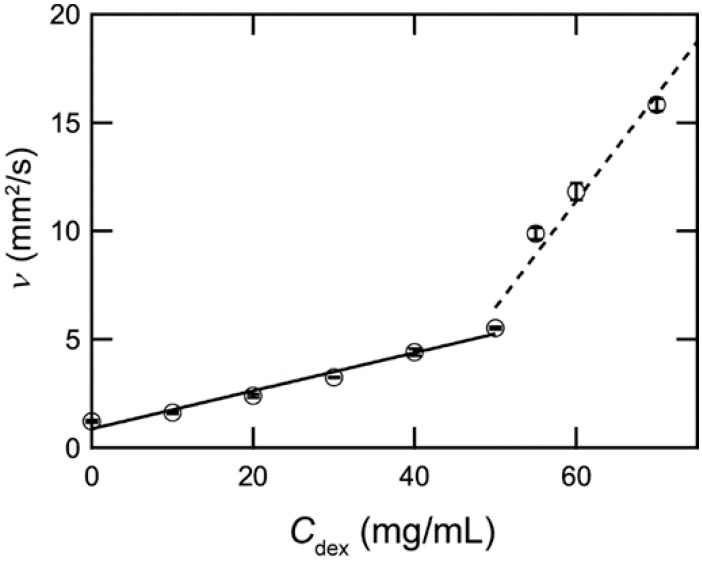
Relationship between *C*_dex_ and ν_in_ a water-based dextran solution. The solid and dashed lines represent the linear regression between a *C*_dex_ ranging from 0–50 and 50–70 mg/mL, respectively. Error bars represent the standard error of the mean calculated from three independent measurements.

Figure 1 shows the relationship between the concentration of DEX *C*_dex_ and kinetic viscosity ν in a water-based dextran solution. *C*_dex_ denotes the concentration of dextran in solution and ν represents kinetic viscosity. ν increases linearly as *C*_dex_ increases. Since the slope changes at 50 mg/mL *C*_dex_, the overlap concentration C* was deduced to be ca. 50 mg/mL. This value was 2.5 times greater than that of a water-based PEG (50 K) mixture, 20 mg/mL [4]. 

**Figure 2:**
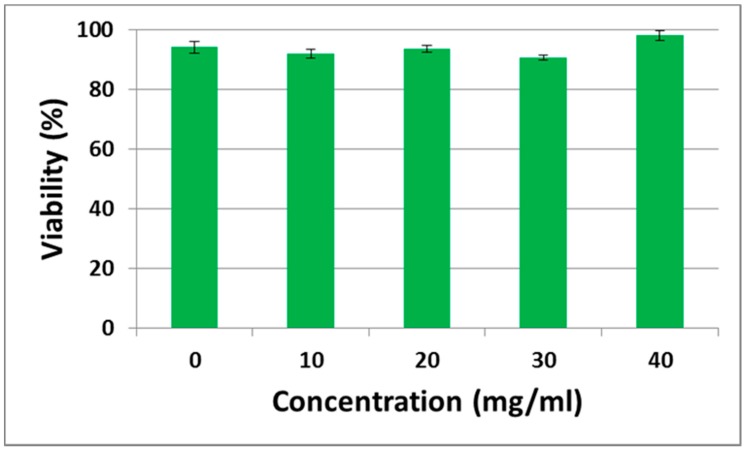
Viability of NMuMG cells. These cells were treated with various concentrations of dextran ranging from 0 to 40 mg/mL. Error bars represent the standard error of the mean calculated from three independent measurements.

To determine the cytotoxic effect of dextran, we adapted a trypan blue exclusion method to identify the proportion of viable cells. As a result, 40 mg/mL of dextran-containing medium was associated with a NMuMG cell viability similar to that of cells cultured in a medium without dextran (Figure 2). This suggests that, even after treatment with laser tweezers, cellular activity in the presence of dextran is maintained, without cytotoxicity.

**Figure 3:**
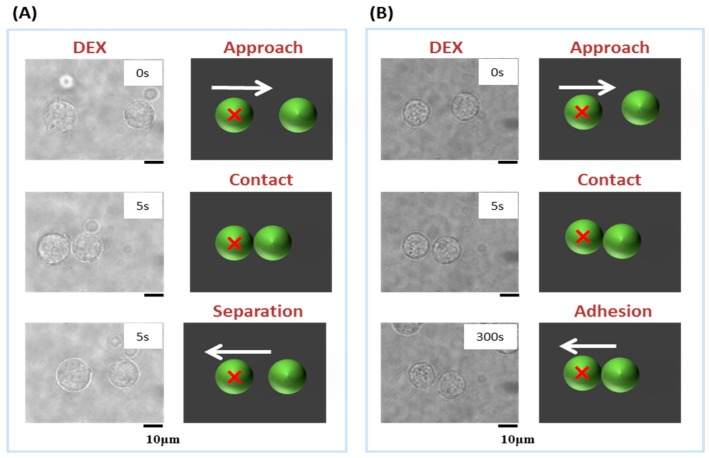

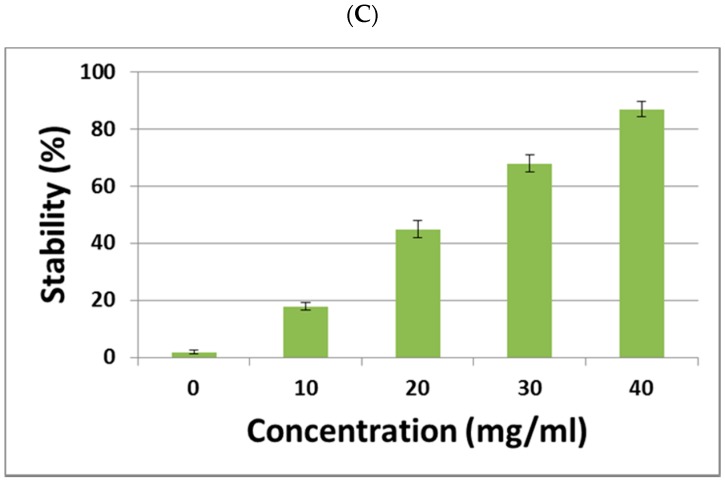
Laser manipulation of a pair of epithelial cells (NMuMG); (**A**) 5 s or (**B**) 300 s in the presence of dextran (40 mg/mL): Spatio-temporal diagram illustrating the process of manipulation (Approach, Contact or Separation (**A**) or Adhesion (**B**)). (**C**) The probability that stable cell–cell contact is maintained through optical transportation for the distance of ca. 5 mm, i.e., the percentage of experimental runs to obtain the result as exemplified in (**B**), where the result as in (**A**) was counted as a failure. Error bars represent the standard error of the mean calculated from three independent measurements.

Figure 3 examines the optical-tweezer-dependent mechanism of cell–cell contact. In this experiment, we applied single-beam optical tweezers. Figure 3A,B show cell adherence induced with optical tweezers for 5 and 300 s, respectively. As shown, 5 s of forced contact was insufficient to induce cell adherence. On the other hand, as reported previously, 300 s of forced contact produced stable cell–cell contact. This suggests that 300 s are required to produce cell adherence and in turn establish 3D cellular assembly. Figure 3C demonstrates the stability of cell–cell contact represented by the percentage of surviving cellular pairs following the indicated actions (approach or contact).

**Figure 4:**
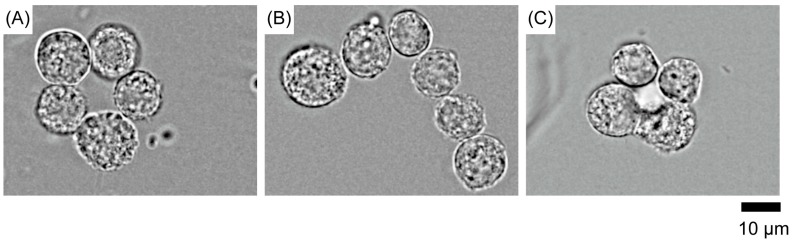
Assemblies of epithelial cells (NMuMG) of various shapes in a medium with DEX (40 mg/mL): the shape of a donut (**A**), letter ‘L’ (**B**), and tetragonal pyramid as an example of 3D cluster (**C**).

Figure 4 demonstrates the formation of various cell morphologies. Here, we applied double-beam optical tweezers.

**Figure 5:**
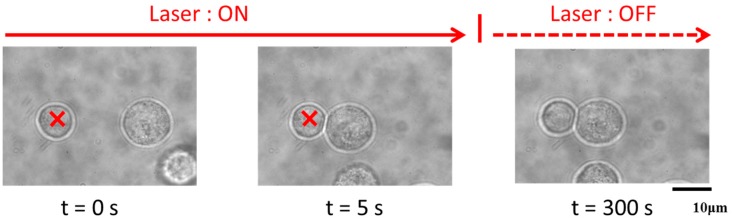
Stable cellular assembly of Neuro2A cells in a dextran (50 mg/mL) medium using optical laser tweezers. The focal point of the laser is marked by the red ‘x’.

Figure 5 confirms the formation of various cell morphologies from Neuro2A mouse brain neuroblastoma cells. In this experiment, we applied single-beam optical tweezers. Neuro2A cells were manipulated with optical tweezers to form various cellular structures. Since undifferentiated Neuro2A cells are used in models of neuronal differentiation [24], 3D assembly of these cells may be useful for modelling neuronal differentiation in 3D cell structures. This system may serve to demonstrate the relationship between 3D cell positioning of undifferentiated neuronal stem cells and neurogenesis. 

## 4. Discussion

The present study successfully extends our recent results on the use of solvable polymers for 3D single-cell-based manipulation. This system adapts an invasive cell assembly method involving optical tweezers and dextran. This dextran-based method is expected to serve as a next-generation 3D single-cell assembly system with future applications in cell biology and regenerative medicine. The results of the present study suggest further advancement of the present method for the formation of stable 3D cellular assemblies through the use of various kinds of solvable polymers, including proteins, polysaccharides, polynucleic acids, etc.

It is widely considered that cell adhesion may be engendered using the congestion or crowding effect [4], which has been attributable to the effect of higher osmotic pressure. Here, it is noted that the effect exerted by a polymer “crowding” solution on living cells is much different from the simple osmotic pressure caused by the addition of small solute molecules. With an increase in osmotic pressure due to the presence of small solutes, cells tend to shrink. However, such osmotic pressure does not directly switch cell–cell interaction from repulsive to attractive. Cell aggregation is a secondary effect caused by an increase in osmotic pressure. In contrast, a solution crowded with macromolecules or large polymer chains causes a so-called depletion effect [4,25,26]. The attractive interaction as a result of the depletion effect by polymer crowding occurs at a length scale on the order of the size of the polymer chain, for example on a scale represented by *R*_g_ for the radius of gyration or *R*_H_ for the hydrodynamic radius. In contrast, as for the osmotic effect of polymers, the increase in osmotic pressure is roughly proportional to the contour length or full-stretch length of the polymer chain, which is proportional to the volume as the product of the contour length and the cross-sectional area of the polymer, being much smaller than the volume occupied by the polymer chain. Thus, in general, the depletion effect is much more significant for polymer chains with a large contour length of more than several tens of nm. The attractive interactions between facing cells due to the entropic depletion force causes flat contact between a pair of cells by creating a gap with the size of the polymer, accompanied by slight deformation of the curvature of the facing side of the membrane on the order of the energy of thermal fluctuation. The occurrence of such a flat gap contributes to the formation of stable cellular contact, by allowing the 2D random walk of individual membrane components, such as membrane proteins and sugar lipids, so as to form attractive contact pairs upon encountering membrane surfaces. The stabilization energy between the facing membranes per unit area due to depletion interaction, ε_α_, is expressed with approximation as [4],

(1) $$\begin{array}{lll} \varepsilon_{\alpha }=-\pi_{P}\left(2d-x\right) & when & x\leq 2d\\ \varepsilon_{a}=0 & when & x>2d, \end{array}$$

where $\pi_{P}$ is the depletion pressure due to the coexisting polymer such as DEX polymer; *d* is the diameter of the polymer in a random coil confirmation; and *x* is the distance between the facing membranes. Thus, the attractive force per surface area due to the depletion effect can be described as,

(2) $$\begin{array}{lll} P_{dep}\approx -\frac{\partial \varepsilon_{\alpha }}{\partial x}=-\pi_{P} & when & x\leq 2d\\ P_{dep}\approx 0 & when & x>2d. \end{array}$$

Meanwhile, the repulsive interactions between a pair of cells floating in solution, such as electric interactions [27], short-range hydration repulsion force [28], and membrane duration [29,30], are inherent properties of a cell membrane [31]. The following relationship has been proposed for repulsive interaction between facing membranes [27]. The positive pressure *P*_rep_ can be represented as,

(3) $$P_{rep}=P_{rep}^{0}\exp\left(-\frac{x}{L}\right),$$

where $P_{rep}^{0}$ is a positive variable that depends on the ionic strength of the medium and the surface potential at the extreme of x=0; and *L* is the characteristic length on the order of 1 nm for a conventional medium for cell culture.

Based on the above arguments regarding the interactions between facing membranes, the net pressure *P*_net_ is the sum of the depletion and repulsive interactions: $P_{net}=P_{dep}+P_{rep}$ [4]. With regard to the experimental conditions for DEX (200 k), the size of the DEX polymer chain in solution, *d*, is regarded as 15–20 nm [32]. We reported that the transmembrane distance is x=10 −15nm in the balance between attractive and repulsive contributions in solutions containing PEG as a crowding polymer [4]. Under similar treatment, we deduced that the width of the gap between facing cells in the present work is of a similar order of magnitude, i.e., x=10−15nm. Thus, when cell–cell contact is achieved in the presence of a crowding polymer, there remains a void space with a width comparable to the size of the polymer between the facing membranes of the adjacent cells. As we discussed in our recent article, the existence of this void space between facing membranes provides the opportunity for the facing membranes to switch repulsive interaction to attractive interaction during cell–cell contact due to dielectric attraction under a focused laser. As a result, stable cell–cell contact could be achieved even after the removal of the coexisting polymer [4]. With regard to the generation of attractive interaction between neighboring cells, a crowding solution environment causes DNA condensation and compaction through the occurrence of the attractive interaction between negatively charged DNA segments [33]. The essential mechanism of attraction is similar to that in our observation of the formation of stable cell–cell contact in PEG solution [4]. Altogether, the overall findings suggest that repulsive single cells can be easily attracted to each other in a medium which contains water soluble polymers such as dextran and PEG, through the depletion effect. This effect facilitates us to construct 3D single cell structures and is, therefore, useful for further applications.

In this study, stable cellular adhesion of NMuMG cells was achieved at concentrations even lower than the overlap concentration C*. The fact that the polymer solution formed stable cell–cell contact below C* is of value because the viscosity of the solution is essentially the same as that of the usual cell culture medium. Under such low viscosity, transportation of individual cells by laser does not encounter any difficulty. While PEG is a synthetic polymer, we have demonstrated for the first time that a natural polymer (dextran) can precipitate cell adhesion and facilitate 3D cellular assembly. In related studies on the use of DEX solution for cell manipulation, Takayama et al. have examined cell printing on a solid substrate. With the use of a dextran solution, they successfully localized cell aggregates at desired positions [34,35]. They reported that the gene expression profile of cells that differentiated after treatment with dextran solution was almost the same as that of cells in the usual medium. In this study, we noted that 10–40 mg/mL of dextran did not have any cytotoxic effect on NMuMG cells. Moreover, since natural compounds like dextran are relatively safe and therefore easily added to the cell culture medium, our dextran-based method may be able to achieve relatively less invasive 3D single-cell assembly.

Cell–cell contact influences the ability of progenitor cells to differentiate [36,37]. Moreover, embryonic stem cells are occasionally cultured as 3D aggregates, known as embryoid bodies. This enhances the differentiation of several cell types. Similarly, neurogenesis can be triggered using sphere-cultured P19 mouse carcinoma cells [38]. Therefore, how these cells are reconstituted in a 3D structure is quite important for proper differentiation. In this regard, our dextran-based 3D cell assembly technique may enable users to control 3D-structure assembly in the hope of achieving novel advances in regenerative medicine.

Cell therapy has received great attention in the field of neuroscience. In the near future, these therapies may be used to treat neurodegenerative diseases such as Parkinson’s disease. In this regard, we were able to make single cell–cell contact using Neuro2A cells. Since neuronal circuits are well organized, it is important to precisely manage the position of neuronal cells and their progenitors. Our 3D single-cell assembly method together with DEX should help to facilitate the reconstitution of damaged neuronal networks. To this end, our approach, which involves the use of optical tweezers, may contribute to the regeneration of well-functioning neuronal systems. Since neuronal systems are composed of many cell types, our next challenge will be to produce lasting cell–cell adhesion between these various cells. Moreover, although we optimized the conditions required for fine control of the position of neuronal progenitor cells in this study, a functional analysis was not performed. Therefore, it will be important to further evaluate our findings using long-term cell culture methods and various functional assays (e.g., single cell transcriptome analysis). To develop functional organoids, which has recently been used as the scientific term to indicate 3D organ-like structures composed of specific cell types or progenitor cells [39,40], it is important to establish an experimental methodology to construct 3D cellular assembly of a large number of cells (above the order of one hundred) with various morphologies. In this regard, we would like to propose the future extension of our study with the hierarchical construction of small cell assemblies (on the order of ten) into a larger assembly with specific shape where each small cell assembly exhibits suitable positioning to generate the specific function as an organoid.

Concluding the present article, we would like to stress the difference between the crowding effect or depletion effect due to macromolecules and the effect of osmotic pressure. The depletion effect induces attractive interaction between neighboring cells through the effect of the exclusion volume by macromolecules on the scale of *R*^3^, where *R* is the radius of a polymer chain as represented by *R*_g_ or *R*_H_. In contrast, the osmotic effect scales with the exclusion volume along the polymer chain, which is much smaller than the scale of *R^3^* for the depletion volume of macromolecules. Additionally, depletion interaction induces attractive interaction between neighboring cells as a primary, direct effect. The present study indicated that depletion interaction is promising for the formation of stable cell–cell contact. We have shown that the natural macromolecule DEX is useful for this purpose, similar to the synthetic polymer PEG. Although there is a large difference in conformational characteristics between DEX and PEG, they result in similar cell–cell attraction, suggesting that the depletion effect can generally be applicable for the construction of stable 3D cellular assemblies. Further extension of this study is expected to contribute to future developments in cellular biology and regenerative medicine. Lastly, we would like to stress that most eukaryotic cells maintain their lives surrounded by crowding body fluids.
